# Challenges in the Use of Artificial Intelligence for Prostate Cancer Diagnosis from Multiparametric Imaging Data

**DOI:** 10.3390/cancers13163944

**Published:** 2021-08-05

**Authors:** Daniele Corradini, Leonardo Brizi, Caterina Gaudiano, Lorenzo Bianchi, Emanuela Marcelli, Rita Golfieri, Riccardo Schiavina, Claudia Testa, Daniel Remondini

**Affiliations:** 1 Department of Physics and Astronomy “Augusto Righi”, University of Bologna, 40127 Bologna, Italy; daniele.corradini3@unibo.it (D.C.); leonardo.brizi2@unibo.it (L.B.); daniel.remondini@unibo.it (D.R.); 2 INFN, Istituto Nazionale di Fisica Nucleare, Sezione di Bologna, 40127 Bologna, Italy; 3 Department of Radiology, IRCCS Azienda Ospedaliero-Universitaria di Bologna, 40138 Bologna, Italy; caterina.gaudiano@aosp.bo.it (C.G.); rita.golfieri@unibo.it (R.G.); 4 Division of Urology, IRCCS Azienda Ospedaliero-Universitaria di Bologna, 40138 Bologna, Italy; lorenzo.bianchi3@gmail.com (L.B.); riccardo.schiavina3@unibo.it (R.S.); 5 Department of Experimental, Diagnostic and Specialty Medicine, University of Bologna, 40138 Bologna, Italy; 6 eDIMES Lab—Laboratory of Bioengineering, Department of Experimental, Diagnostic and Specialty Medicine (DIMES), University of Bologna, 40138 Bologna, Italy; emanuela.marcelli@unibo.it; 7 IRCCS Istituto delle Scienze Neurologiche di Bologna, 40139 Bologna, Italy

**Keywords:** prostate cancer, AI, mp-MRI, PI-RADS, public databases

## Abstract

**Simple Summary:**

Prostate Cancer is one of the main threats to men’s health. Its accurate diagnosis is crucial to properly treat patients depending on the cancer’s level of aggressiveness. Tumor risk-stratification is still a challenging task due to the difficulties met during the reading of multi-parametric Magnetic Resonance Images. Artificial Intelligence models may help radiologists in staging the aggressiveness of the equivocal lesions, reducing inter-observer variability and evaluation time. However, these algorithms need many high-quality images to work efficiently, bringing up overfitting and lack of standardization and reproducibility as emerging issues to be addressed. This study attempts to illustrate the state of the art of current research of Artificial Intelligence methods to stratify prostate cancer for its clinical significance suggesting how widespread use of public databases could be a possible solution to these issues.

**Abstract:**

Many efforts have been carried out for the standardization of multiparametric Magnetic Resonance (mp-MR) images evaluation to detect Prostate Cancer (PCa), and specifically to differentiate levels of aggressiveness, a crucial aspect for clinical decision-making. Prostate Imaging—Reporting and Data System (PI-RADS) has contributed noteworthily to this aim. Nevertheless, as pointed out by the European Association of Urology (EAU 2020), the PI-RADS still has limitations mainly due to the moderate inter-reader reproducibility of mp-MRI. In recent years, many aspects in the diagnosis of cancer have taken advantage of the use of Artificial Intelligence (AI) such as detection, segmentation of organs and/or lesions, and characterization. Here a focus on AI as a potentially important tool for the aim of standardization and reproducibility in the characterization of PCa by mp-MRI is reported. AI includes methods such as Machine Learning and Deep learning techniques that have shown to be successful in classifying mp-MR images, with similar performances obtained by radiologists. Nevertheless, they perform differently depending on the acquisition system and protocol used. Besides, these methods need a large number of samples that cover most of the variability of the lesion aspect and zone to avoid overfitting. The use of publicly available datasets could improve AI performance to achieve a higher level of generalizability, exploiting large numbers of cases and a big range of variability in the images. Here we explore the promise and the advantages, as well as emphasizing the pitfall and the warnings, outlined in some recent studies that attempted to classify clinically significant PCa and indolent lesions using AI methods. Specifically, we focus on the overfitting issue due to the scarcity of data and the lack of standardization and reproducibility in every step of the mp-MR image acquisition and the classifier implementation. In the end, we point out that a solution can be found in the use of publicly available datasets, whose usage has already been promoted by some important initiatives. Our future perspective is that AI models may become reliable tools for clinicians in PCa diagnosis, reducing inter-observer variability and evaluation time.

## 1. Introduction

Prostate Cancer (PCa) is one of the most common types of cancer among men, causing thousands of deaths every year worldwide [1]. The more effective weapon against this disease is the early detection of clinically significant tumor lesions. Confirming PCa presence and staging requires the inspection of the gland tissue by pathologists. Indeed, for many years, systematic sampling of the whole prostate because of rising PSA (Prostate Specific Antigen) has represented the gold standard to diagnose PCa, due to a lack of accurate imaging modality to detect PCa foci early. As a result, men without cancer often undergo unnecessary systematic prostate biopsies with a potential risk of complications (mainly infections), and clinically indolent cancers are often detected, but biologically aggressive cancers are sometimes missed [2].

So far, the best alternative for PCa staging is multiparametric Magnetic Resonance Imaging (mp-MRI), an ensemble of non-invasive imaging techniques, such as T2-weighted (T2W), Diffusion-weighted (DWI), and Dynamic Contrast Enhancement (DCE) imaging [3]. Moreover, mp-MRI is an essential tool for a targeted prostate biopsy that significantly increases the detection of clinically significant PCa in different settings [4], for surgical planning of nerve sparing approach [5], and for risk assessment during active surveillance protocol [6]. Recently, the technique of prostate biopsy called “fusion biopsy”, which allows a co-registration between MRI and echographic images, has drastically improved the detection rate of prostate biopsy thanks to the accuracy of mp-MRI [4].

For determining whether a lesion can be labeled as clinically significant or clinically non-significant, a guideline has been drawn up: The Prostate Imaging Reporting and Data System (PI-RADS). PI-RADS version 2.1 [7] is a standard for reading mp-MR images to risk-stratify PCa by assigning a score to the suspected lesions index that ranges from 1 to 5, from benign to malign PCa, depending on the lesion’s aspect and localization [8]. Despite the attempt to standardize image interpretation with the use of PI-RADS guidelines, inter-observer variability due to the reader experience is still a current issue, especially when the lesions show an intermediate level of aggressiveness, labeled with the equivocal PI-RADS score of 3 [9]. In those cases, the aggressiveness of the lesion can be assessed only by histopathology. To reduce the number of false-positive, and hence unnecessary, biopsies, an improvement in reading MR images is desired. Thus, researchers are investigating high-specificity and more standardized approaches to map the histopathology outcomes through radiomics, an approach based on the analysis of quantitative features extracted from images [10,11]. In what follows, we will discuss the recent achievement and what could be improved in PCa characterization using Artificial Intelligence (AI) methods.

## 2. AI in PCa Characterization

AI has become a very widely used and trendy term that can be used to refer to different approaches. Here we follow the common framework for which AI includes methods such as Machine Learning and Deep Learning. AI tools can help PCa diagnosis in many aspects, such as in prostate gland volume segmentation, lesion segmentation, detection, and characterization [12,13], and it has also been developed for applications in robotic surgery both for prostate cancer [14] and kidney cancer [15], digital pathology, and genomics [16]. However, one of the most challenging tasks is to accurately stage lesions aggressiveness, in particular classifying between not clinically significant tumors and clinically significant PCa. The threshold between indolent and aggressive lesions is usually set at a Gleason Score (GS) of 7 that can be obtained by the Gleason Grades (GG) 3 + 4 or 4 + 3. However, as we will see below, there is no broad consensus among the studies.

In the literature, studies that classify different levels of aggressiveness implement various AI techniques, both the traditional Machine Learning (ML) algorithm and the more advanced methods of Deep Learning (DL), in particular based on Convolutional Neural Networks (CNN), particularly tailored for the processing of imaging data. Discarding all research that just included the classification between malign and benign lesions, we selected a total of 18 studies [17,18,19,20,21,22,23,24,25,26,27,28,29,30,31,32,33,34], including one study from 2015 [22], three from 2017 [17,18,27], two from 2018 [24,28], nine from 2019 [19,25,26,29,30,31,32,33,34], and three from 2020 [20,21,23].

We checked how the studies obtained their reference standard, that is the GS or GG from the histological findings. Although prostatectomy is more precise as the whole mount of the gland is inspected, it is used just in three studies [27,32,33] and partially in one paper [21]. The rest of the studies adopted systematic transrectal ultrasound-guided, MR-guided, or MRI/US- fusion biopsy for acquiring their gold standards.

To define the potential value of AI methods for PCa stratification, the key point in research is to evaluate the performance of new methods for discrimination between indolent and clinically significant cancers. The threshold has been set correspondingly to an intermediate risk of cancer historically corresponding to GS = 2 + 4, 4 + 2, 3 + 4, 4 + 3. Nevertheless, the most recent histological evaluation of prostate specimen considers a threshold of GS = 7 as reliable considering the finding corresponding to GS = 2 + 4 and GS = 4 + 2 not reliable. This is the reason why most of the recent studies tried to separate lesions with GS ≥ 7 and GS < 7. Only one [31] set the threshold at GS = 8. Conversely, Litjens et al. in 2015 [22] shifted the threshold towards lower values of GS, namely, they separated GS = 3 + 3 against the higher scores (including GS = 2 + 4). Similarly, also in [34], they divided lesions with the same GS but different GGs, i.e., they considered GS = 3 + 4 within the indolent group and GS = 4 + 3 within the aggressive group. Noticeably, only two studies performed a variety of classification tasks [25,29]. Specifically, Jensen et al. [29] performed binary classification separating lesions belonging to one GG versus the others, trying different GGs combinations. The best performance (AUC 0.98, sensitivity 1, specificity 0.95) was obtained for GG 3 vs. the rest for lesions in the peripheral zone while the worst results were reported for GG 1 + 2 vs. rest for lesions in the transition zone (AUC 0.83, sensitivity 0.86, specificity 0.80). Differently, Cao et al. separated low- and high-grade tumors trying different GS thresholds. Namely, they classified lesions with GS ≥ 7 vs. GS < 7 and GS ≥ 4 + 3 vs. GS ≤ 3 + 4 achieving an AUC of 0.81 and 0.79, respectively, showing a slight decrease when attempting to further stratify the lesions with intermediate risk. Moreover, they tried to increase the thresholds at GS 8 and 9 but the performances dropped to AUC 0.67 and 0.57, respectively. All the classification tasks are reported in Table S1.

**Figure 1:**
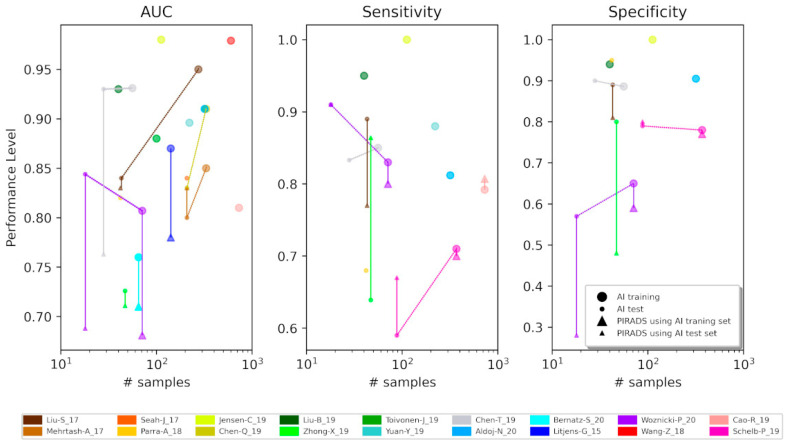
Scatter plots showing the trends of the classification performance (AUC, Sensitivity, and Specificity) against the number of samples. When different models were implemented, the best results have been picked. The bigger symbols represent the performance achieved by training sets, while the smaller ones are by test sets. The round-shaped dots indicate the performance level obtained through AI models while the triangle-shaped dots by PI-RADS score. The two are linked by a bold line. Conversely, dotted lines connect performance evaluated with training and test sets obtained from the same AI model. Each color represents a specific study, as reported in the legend below the plots. In case this paper is read in html format, when a point is mouse hovered, a tip will pop up with the name of the paper, the dataset (training or test set), the number of samples, the performance (AUC, sensitivity or specificity value), the classification task, and the method description (AI model or PI-RADS) [see Table S1 if the html is not available].

The goodness of the AI models can be evaluated not only by the overall performance but also by comparing it with the PI-RADS score. However, not all the considered studies reported the respective PI-RADS results. Moreover, one study [22] used only PI-RADS version 1, two studies [18,26] used both version 1 and 2, and just one [23] adopted the latest version 2.1. In Figure 1, the triangle-shaped dots show the performance achieved by radiologists, which are directly linked by a line with the AI performance, indicated by round-shaped dots. The size of the markers relates to the different datasets used to define the performance of methods: The biggest refers to the training set (the set of samples used to find the parameters of the model, which in our context, includes the sub-set for validation) and the smallest refers to the test set (the set of samples used to provide an unbiased evaluation of the final model on unseen data). When using the AUC metric, almost all the AI models achieved a higher performance with respect to the PI-RADS evaluation. The best improvement was obtained by Woznicki et al. [23] where the AUC from 0.69 is raised to 0.84 when applying the model to a test set. In addition, Chen et al. [19] showed an impressive increase from 0.76 to 0.93, using a test set as well. In [17,32], the AI approach did not get much better than PI-RADS, while [18] was the only study where PI-RADS achieved a slightly higher level of performance: An AUC of 0.83 against the 0.80 of the CNN. Extracting the AUC metric only for those studies that considered prostatectomy as the reference standard, AI approaches obtained very good performances: in [24,25,32,33], AUC ranged from 0.81 to 0.95. The requirement of prostatectomy as a gold standard will sensibly reduce the number of data to work with AI, but these few results show that the proper gold standard used as reference can help the performance of AI models given the high AUC obtained.

When considering the sensitivity, only Liu et al. [17] AI showed a much better outcome (0.77 vs. 0.89), while the others obtained comparable results [23,25], or even worse, like in [26,32] with a sensitivity of 0.67 vs. 0.59 and 0.86 vs. 0.63, respectively. Sensitivity allows one to extrapolate the percentage of missed lesions (that corresponds to the percentage of the false negative cases). Only a few studies comment on this score giving possible explanations for false negative such as the presence of small and subcapsular lesions [35]. As far as the specificity is concerned, the results are much less controversial, with similar results obtained in [26] and better AI performances achieved in [14,19,28], where the specificities on the test set of PI-RADS and AI are 0.81 vs. 0.89, 0.48 vs. 0.8, and 0.28 vs. 0.57, respectively.

Usually, AI models achieved higher or comparable results with respect to the radiologists’ outcomes. However, sometimes PI-RADS seems to be more performant, a sign that AI methods still have some limitations. In Zhong et al. [32], the authors reported many possible reasons for explaining the weakness of their model. Interestingly, they stressed the importance of implementing 3D AI models for exploiting the full potential of mp-MRI. In the considered studies, only [17,23,32] adopted a 3D CNN.

Noteworthy, some studies also included PI-RADS scores as part of the AI implementation, for example using the scores as one of the features in ML algorithms [21] or combining AI and PI-RADS outcomes to better discriminate clinically and not clinically significant PCa [22]. In the first case, using some hand-crafted radiomic features together with PI-RADS scores could improve the predictive performance when properly combined. In the second case, the authors showed that combining the CAD (Computer-Aided Diagnosis) results with PI-RADS scores improved power prediction with respect to the PI-RADS alone, specifically from 0.78 to 0.87.

As far as the type of AI model implemented in the studies is concerned, 8 out of 18 studies [19,21,22,23,28,29,31,33] used a ML approach while the remaining 10, [17,18,20,24,25,26,27,30,32,34] implemented a CNN for the classification tasks.

Although more samples were used for training the CNNs than the ML algorithms (i.e., 367 against 86 on average), the two approaches did not show significant differences, as the overall AUC achieved was 0.86 ± 0.07 and 0.88 ± 0.07, respectively. A more marked difference between ML and DL techniques is presented by Cuocolo et al. [36], where the ML classifiers performed slightly better, with a pooled AUC of 0.90 ± 0.02, while the DL methods just achieved an average AUC of 0.78 ± 0.04.

Therefore, the available results show that the use of AI approaches still presents some weaknesses. The major problems can be resumed in two main points: Overfitting due to the adoption of small datasets and the lack of standardization/reproducibility of acquired imaging data. These issues are mainly due to the data generation procedure. In general, to obtain high-quality mp-MR images for AI models, the following general steps are followed: (1) MR multi-sequence acquisition; (2) application of suitable preprocessing steps to reduce noise and variability; (3) lesions segmentation and level of aggressiveness identification. This procedure is cost- and time-consuming, explaining the reduced number of images usually available. Besides, each step can be affected by great variability and may be case-dependent, leading to a low level of standardization among the studies.

### 2.1. Overfitting

It is well known that AI methods, and in particular DL algorithms, require many data to efficiently train the net and properly generalize to test data. As shown above, it is hard to gather a well-sized dataset to avoid overfitting. For example, in the cited papers, the mean number of patients is 155 (min: 40 [31], max: 417 [25]). However, to fully grasp the issue of the lack of samples, one should consider the number of lesions used for training the AI models. In most of the papers, not only the dominant lesions but all the lesions within the prostate were considered except for a few experiments [21,23,31]. Nevertheless, this difference seems not to affect the performance of the methods. However, to fully grasp the issue of the lack of samples one should consider the number of lesions used for training the AI models. The average number of lesions used in the training set is 242 (min: 40 [31], max: 728 [25]) and in the test set is 99 (min: 18 [23], max: 208 [18]. These lesions are not always divided equally among the classes; in fact, only in [21,26,31,32,35,37] do the classes contain a similar number of samples. For dealing with unbalanced classes, few studies [18,19,20,28,30,32] used different techniques, such as SMOTE (synthetic minority oversampling technique) or a simple re-sampling by using data augmentation.

Considering the overall distribution of the lesion localization in all 18 studies, most of the lesions are found in the Peripheral Zone (PZ) (58.35%), then in the Transition Zone (TZ) (24.96%), and the Anterior Fibromuscolar Stroma (AFS) (12.58%). A small component is also found in the central zone (CZ) and in the seminal vesicle (VS), which together count for less than 5% of the total. From a clinical point of view, it is also important to understand the place of the lesion within the prostate gland, because depending on the zone, it can be harder to identify and characterize its aggressiveness, especially for non-experienced readers. The only study that performed different classifications based on the lesion localization is [30], which investigated classifications separating the lesions from PZ and TZ/AFS and achieved an average AUC of 0.92 ± 0.05 and 0.88 ± 0.04, respectively. Although lesions belonging to TZ are usually more complicated to identify and characterize, even for AI models, these results are promising.

Interestingly, a decreasing trend of the performance against the number of samples (lesions) used for testing the AI model was noticed as shown in Figure 1. When using AUC and sensitivity, the respective coefficient of correlation is −0.14 and −0.78, respectively, confirming the negative trend. This is a sign of overfitting because if one trains the algorithm with few samples that do not cover all the variability of the lesion types, grades, and locations, when using a relatively big test set, it can happen that the classifier is not able to recognize the new cases. Weirdly, in the case of specificity and sensitivity also for the training set, there is a negative correlation between samples and performance, but this could depend on many other factors, such as the model implemented or the quality of the data. In addition, most of the studies did not adopt an independent test set, adopting a cross-validation approach using all the data available.

There are mainly two strategies to solve overfitting if more data cannot be gathered: Data Augmentation (DA) and Transfer Learning (TL) [37,38]. DA multiplies the images at hand using a series of operations, such as rotation, translation, cropping, blurring, shearing, scaling, etc., to increase the variability within the training dataset and thus the generalization ability of the AI model. As reported in Table S1 in Supplementary Materials, most of the studies adopted DA for dealing with the small sample size, excepted the few in which it is not explicitly reported [21,23,24,29,31,33,38]. On the other hand, when adopting TL, the AI model is partially pre-trained using a larger dataset, even of different image types. Subsequently, when the neural network is trained with the mp-MR images, some of the weights are fixed, allowing only the outer layers to be updated during the learning process. Well-known datasets created for visual object recognition are ImageNets, used in [30,34], and CIFAR-10, used in [32], which include thousands of annotated images divided into different categories.

### 2.2. Standardization and Reproducibility

We have already stressed the complex procedures behind mp-MR image acquisition. Here we are going to focus on the protocol variability among the different studies. Firstly, it is important to show the data source heterogeneity, which is from how many centers the images have been acquired. Most of the studies obtained the mp-MR sequences from a single institution, only one [28] came from two institutions, and two studies [24,34] used their own institution dataset together with publicly available images (Prostate-X [37]). Therefore, so far, the adoption of multi-center data is quite limited. Even within the same institution, different hardware setups for the MR acquisition can be used. For instance, most of the studies used more than one scanner, except in [19,21,26,31,33]. The magnetic field strength of the scanner was always 3T, except in [31] where it was 1.5T or in [28] where both scanners at 3T and 1.5T were used.

As far as coils are concerned, there is also variability in the choice of the endorectal coil (ERC), which was only adopted in part of the acquisitions in [25,29,32]. Some studies have reported a higher quality of MR image acquired with ERC [39]. However, the overall staging accuracy is usually not significantly different [39,40,41] even though in [41] it has shown a greater performance in revealing cancer at an intermediate level of aggressiveness (GS = 3 + 4). Nonetheless, in the considered studies, the overall performance when using an ERC was comparable to the cases with another type of coils, with an AUC of 0.78 ± 0.05 and 0.83 ± 0.06, respectively. Although the ERC increases the signal-to-noise ratio, artifacts due to its geometry and susceptibility variations could interfere with this benefit.

When dealing with multi-sequence, multi-center, multi-scanner, or multi-coil data, it is fundamental to apply some preprocessing steps, such as the normalization of the intensities for reducing variability and registration of the images acquired with different MR sequences into the same space. Only a few studies did not use [21] or did not report any preprocessing steps [19,23,31,37]. Table 1 summarizes the information reported above about the datasets and the MR image acquisitions and processing.

Mp-MRI is composed of three sequences: T_2_W, DWI, DCE. However, some studies used just T_2_W-DWI [20,24,25,26,27,34,36] or DWI-DCE [18,28] or only DCE [31]. Interestingly, more than half of the studies used DCE and not always together with the other two sequences. Furthermore, in [42], the higher AUC achieved with a model including DCE has been shown. Therefore, DCE plays an important role in AI applications. Conversely, DCE has a secondary role in the PI-RADS evaluation of clinically significant PCa [43]. However, when used together with T_2_W and DWI images (depending on the lesion location), it can be useful for detecting small lesions and characterizing indeterminate findings [42,44].

Commenting on all the chosen protocols parameters is out of the scope of this paper. However, we would like to report the papers that did not give any information on the in-house acquired images [34] or that reported a minimal description [29,30]. Others [17,18,27] did not report any information but they used a public dataset whose protocols can be found easily elsewhere. There is a specific parameter of the mp-MRI protocol whose choice is a remarkable source of variability that is the high b-value of DWI. In agreement with the PI-RADS 2.1 guidelines [7], for acquiring ADC maps, the DWI sequences of the mentioned studies have at least two values: One low b-value from 0 to 50 s/mm^2^ (although the 50–100 s/mm^2^ range is preferred) and the other from 800 to 1000 s/mm^2^. Optionally, intermediate values are also added, ranging from 100 to 500 s/mm^2^. Interestingly, in [28], a high b-value of 1400 s/mm^2^ for ADC maps was used. Sometimes, a DWI sequence with a higher b-value was joined to the image dataset, which ideally should be above 1400 s/mm^2^ [7]. Nonetheless, except in [28,30], the other studies used a high b-values of 800 s/mm^2^.

For the aim of standardization, it is crucial to use the same format for the mp-MR images. The standard for managing and sharing medical images is Digital Imaging and Communications in Medicine (DICOM). As it can be seen in Table 1, the DICOM format was adopted by the studies that used the public dataset Prostate-X [17,18,20,24,27,29,30,34] and just from two institution datasets [21,28]. The other studies did not report this information. Considering the studies that used institution data, only one [33] made the images available upon request. The same study was also the only one that shared the custom-made software of the analysis in a hosting service platform (GitHub). However, some of the studies reported basic information on the software packages used in the analysis pipeline, while others did not give any information [17,22,24,30,31,34]. Disappointingly, sharing data and code is not a common behavior, strongly limiting the possibility of comparative or integrated multi-centric studies.

The lack of standardization affects not only MR image acquisition and analyses but also concerns the reference standard to which images are compared. A standard criterion to consider the clinical significance of prostate cancer has to be shared to facilitate the process of validation of the algorithm deputed to automate lesion classification. A comparison between different AI approaches can be facilitated even with the choice of considering all the lesions present in each organ with respect to considering only the dominant t lesion for each patient.

## 3. The Role of Public Databases

A solution to the problems reported in Section 2.1 and Section 2.2, namely overfitting and lack of standardization/reproducibility, can be a more widespread utilization of publicly available databases. However, only six studies [17,18,24,27,29,30] used public datasets, and two adopted both institution and public images [25,36]. Interestingly, the AI models implemented in the two above-mentioned groups achieved a mean AUC of 0.87 ± 0.06 and 0.93 ± 0.06 using, on average, 282 and 411 lesions, respectively. On the other hand, the studies that only trained their models with institution data [19,21,22,23,25,26,28,31,32,33] achieved a mean AUC of 0.84 ± 0.07 with 184 lesions on average. Thus, using the larger public databases may improve the overall AI performance, and increase their robustness towards new data.

So far, a recent review [45] identified 42 publicly available, patient-centered PCa datasets, but only 8 contain mp-MR image data. All eight datasets (including Prostate-X) are stored in The Cancer Image Archive (TCIA) [46]. TCIA is a large-scale and open-source repository of high-quality images, often supported with genomic, proteomic, and clinical data to investigate cancer phenotype correlates, hence enabling more personalized medicine. TCIA is built following the pillars of the data sharing given by the FAIR principle [47] guidelines to ease the data re-usability. Two of the PCa datasets in TCIA are part of the Quantitative Imaging Network (QIN) [48]. One of the aims of QIN is to help researchers to standardize protocols and procedures and benchmark analysis tools and image biomarkers both for clinical decision-making and prognosis.

In response to the demand for more integration between clinical information and imaging data, another project has been initiated, the Prostate Medical Intelligence System Enterprise-Clinical, Imaging, and Pathology (PROMISE CLIP) [49].

Another promising EU-founded project for boosting the accuracy of AI models in PCa characterization is the ProCancer-I (https://www.procancer-i.eu/, accessed on 20 December 2020). The main purpose of this high-achieving initiative is to develop a platform for storing a large collection of MR images and robust AI implementation to improve tumor staging and hence helping to choose more suitable treatments.

Within these initiatives, it would be desirable that a greater quantity of data would have prostatectomy as a reference standard because this will assure robustness to the models for automatic classification of PCa.

## 4. Conclusions

Although the research community is changing towards better practices in data acquisition, classification, and analysis, many efforts are still required. AI for PCa characterization needs great work in the direction of harmonization of data, for example, it was promoted in the last years by the neuroscience community [50,51]; this could encourage the use of public databases and future studies to combine images coming from different datasets, acquired with heterogeneous MR platforms and protocols. A fundamental contribution will be provided by the very recent international initiatives to integrate multi-centric databases, allowing larger studies and the identification of critical issues in data integration and harmonization in this field.

As we have seen, the AI models performed classifications between not clinically significant and clinically significant lesions with relatively good performance, and sometimes they even outperformed PI-RADS outcomes, suggesting that AI can be a tool to overcome high inter-reader variability or possible lack of reader experience, specifically in the dubious zones like the TZ. However, most of the datasets contained few images that just partially covered all the PCa variability, e.g., given by aspect and localization, leading to a high risk of overfitting. Moreover, the attempt to systematically study how AI models may improve the understanding of the relationship between histopathology findings and mp-MR images, especially for lesions with the ambiguous PI-RADS score 3, is still really limited. Besides, only a few studies try to adopt more standardized approaches or to make their protocols and analysis more reproducible and comparable. In addition, the sharing of processing and analysis pipelines will be a crucial issue, to compare methodologies and identify the weakest points in terms of generalization to new data or specific issues associated with different anatomic regions.

Moreover, considering the recent introduction of PSMA (Prostate Specific Membrane Antigen) PET (Position Emission Tomography) imaging with very promising results in terms of detection PCa both for staging [52] and restaging proposal [53,54], some authors have proposed to combine the anatomic precision of MRI and functional information of PSMA PET by PSMA PET/MRI with the aim to improve the accuracy of detection of PCa foci within the prostate and the identification of nodal metastases. If these promising results are confirmed, multimodal imaging modalities comprising metabolic imaging [55] could give a wide spectrum of features for AI models. Thus, the hybrid PSMA PET/MRI may be the objective of further development of AI methods based on CNN to help radiologists and nuclear medicine physicians with better PCa diagnosis based on high-quality and high-complexity novel imaging. An improvement in terms of the availability of high-quality mp-MR images will enable the development of the promising 3D CNNs [56], using 3D (isotropic) MR acquisitions, to take advantage of the whole lesion structure and aspect to accurately predict its aggressiveness. Noticeably, DL techniques are less prone to pre-processing steps and do not require feature extraction, intrinsically provided by the CNN architecture. Even if the training step is usually quite time-consuming, once the network is ready, an image can be classified very rapidly, typically with a reduced computational burden as compared to the training phase. Considering the enormous amount of time needed by radiologists to analyze mp-MR images, it is surely worthy to keep improving the AI contribution to diagnosis. Hopefully, once the AI models trained to detect and stage PCa lesions with mp-MR images are highly performant and reliable, they will be applied in a clinical setting as a further powerful tool for fighting PCa.

## Figures and Tables

**Table 1:** Table reporting general information on datasets (and their format), MR sequences, scanners, coils, and the image preprocessing (normalization or registration) methods.

Paper	Dataset	MR Sequence	Hardware Setup	Image Processing
Liu, S. 2017 [17]	ProstateX (DICOM)	T_2_W, DWI, DCE	Siemens (Magnetom Trio and Skyra) at 3T without ERC	Registration
Mehrtash, A. 2017 [18]	ProstateX (DICOM)	DWI, DCE	Siemens (Magnetom Trio and Skyra) at 3T without ERC	Normalization
Seah, J. 2017 [27]	ProstateX (DICOM)	T_2_W, DWI, DCE	Siemens (Magnetom Trio and Skyra) at 3T without ERC	Normalization
Parra, A. 2018 [28]	2 institutions (DICOM)	DWI, DCE	(I) Siemens and General Electric at 3T with external pelvic coil; (II) Siemens, Philips, General Electric at 3T and 1.5T with ERC	Registration, Normalization
Jensen, C. 2019 [29]	ProstateX (DICOM)	T_2_W, DWI, DCE	Siemens (Magnetom Trio and Skyra) at 3T without an ERC	Normalization
Chen, Q. 2019 [30]	ProstateX (DICOM)	T_2_W, DWI, DCE	Siemens (Magnetom Trio and Skyra) at 3T without an ERC	Normalization
Liu, B. 2019 [31]	1 institution	DCE	General Electric (Signa Excite II) at 1.5T	N/A
Zhong, X. 2019 [32]	1 institution	T_2_W, DWI, DCE	Siemens (Trio, Verio, Prisma or Skyra) at 3T with pelvic phased-array coil with or without ERC	Normalization
Toivonen, J. 2019 [33]	1 institution	T_2_W, DWI	Philips (Ingenuity) at 3 T with 32 channel cardiac coils	Normalization
Yuan, Y. 2019 [35]	(I)1 institution (II) ProstateX (DICOM)	T_2_W, DWI	(I) N/A; (II) Siemens (Magnetom Trio and Skyra) at 3T without ERC	Normalization
Chen, T. 2019 [19]	1 institution	T_2_W, DWI	Philips (Intera Achieva) at 3T with 32-channel body phased-array coil	N/A
Aldoj, N. 2020 [20]	ProstateX (DICOM)	T_2_W, DWI, DCE	Siemens (Magnetom Trio and Skyra) at 3T without ERC	Registration Normalization
Bernatz, S. 2020 [21]	1 institution (DICOM)	T_2_W, DWI, DCE	Siemens (Magnetom Prisma FIT) at 3T with 32-channel body coil and spine phased-array coil	Limited image manipulation
Litjens, G. 2015 [22]	1 institution	T_2_W, DWI, DCE	Siemens (Trio or Skyra) at 3T without ERC	N/A
Woznicki, P. 2020 [23]	1 institution	T_2_W, DWI	Siemens (Magnetom Skyra and Trio) at 3T with pelvic phased-array coils	N/A
Wang, Z. 2018 [24]	(I)1 institution (II) ProstateX (DICOM)	T_2_W, DWI	(I) Siemens (Magnetom Skyra) at 3T (II) Siemens (Magnetom Trio and Skyra) at 3T without ERC	Registration
Cao, R. 2019 [25]	1 institution	T_2_W, DWI	Siemens (Trio, Skyra, Prisma, Verio) at 3T with and without ERC	Registration Normalization
Schelb, P. 2019 [26]	1 institution	T_2_W, DWI	Siemens (Prisma) at 3T with standard multichannel body coil and integrated spine phased-array coil	Registration

## Supplementary Materials

The following are available online at https://www.mdpi.com/article/10.3390/cancers13163944/s1, Table S1: Table reports the binary classification tasks in the form (class 1 vs. class 2) and the AI models specifying if DL or ML and the anti-overfitting methods (Data Augmentation or Transfer Learning) adopted for the 18 papers considered, named using the first author and the year of publication.
