# Supplementary material for: Challenges in the Use of Artificial Intelligence for Prostate Cancer Diagnosis from Multiparametric Imaging Data
Source: Cancers (Basel). 2021 Aug 5;13(16):3944. doi: 10.3390/cancers13163944 (PMC8391234; doi:10.3390/cancers13163944)
Supplement: Supplementary file 1 [file cancers-13-03944-s001.zip › cancers-1298906-supplementary.pdf]

Supplementary Materials: Challenges in the Use of Artificial Intelligence for Prostate Cancer Diagnosis from Multiparametric Imaging Data

Daniele Corradini, Leonardo Brizi, Caterina Gaudiano, Lorenzo Bianchi, Emanuela Marcelli, Rita Golfieri, Riccardo Schiavina, Claudia Testa and Daniel Remondini

Table S1. Table reports the binary classification tasks in the form (class 1 vs. class 2) and the AI models specifying if DL or ML and the anti-overfitting methods (Data Augmentation or Transfer Learning) adopted for the 18 papers considered, named using the first author and the year of publication.

Paper	Classification Task	AI model
Liu, S. 2017 [17]	(GS ≥ 7 vs. GS < 7)	CNN + DA
Mehrtash, A. 2017 [18]	(GS ≥ 7 vs. GS < 7)	CNN + DA
Seah, J. 2017 [27]	(GS ≥ 7 vs. GS < 7)	CNN + DA + TL
Parra, A. 2018 [28]	(GS ≥ 7 vs. GS < 7)	ML + DA
Jensen, C. 2019 [29]	PZ: (GG1 vs. rest), (GG2 vs. rest), (GG1 + 2 vs. rest), (GG3 vs. rest), (GG4 + 5 vs. rest)<br>TZ + AFS: (GG1 vs. rest), (GG2 vs. rest), (GG1 + 2 vs. rest), (GG3 vs. rest), (GG4 + 5 vs. rest)	ML
Chen, Q. 2019 [30]	(GS ≥ 7 vs. GS < 7)	CNN + DA + TL
Liu, B. 2019 [31]	(GS ≥ 8 vs. GS < 8)	ML
Zhong, X. 19 [32]	(GS ≥ 7 vs. GS < 7 + false positive)	CNN + DA + TL
Toivonen, J. 2019 [33]	(GS ≥ 7 vs. GS < 7)	ML
Yuan, Y. 2019 [35]	(GS = 4 + 3,4 + 4,3 + 5,5 + 3 vs. GS = 3 + 3,3 + 4)	CNN + DA + TL
Chen, T. 2019 [19]	(GS ≥ 7 vs. GS < 7)	ML + DA
Aldoj, N. 2020 [20]	(GS ≥ 7 vs. GS < 7)	CNN + DA
Bernatz, S. 2020 [21]	(GG ≤ 2 vs. GG ≥ 3)	ML
Litjens, G. 2015 [22]	(GS = 2 + 4,3 + 4,2 + 5,3 + 5 or any with 4 or 5 components vs. GS ≤ 3 + 3 and benign lesions)	ML
Woznicki, P. 2020 [23]	(GS ≥ 7 vs. GS < 7)	ML and CNN
Wang, Z. 2018 [24]	(GS ≥ 7 vs. GS < 7)	CNN
Cao, R. 2019 [25]	(GS ≥ 7 vs. GS < 7), (GS ≥ 4 + 3 vs. GS ≤ 3 + 4), (GS ≥ 8 vs. GS < 8), (GS ≥ 9 vs. GS < 9)	CNN + DA
Schelb, P. 2019 [26]	(GS ≥ 7 vs. GS < 7)	CNN + DA

Legend: GG: Gleason Grade; GS: Gleason Score; CNN: Convolutional Neural Network, DA: Data Augmentation; TL: Transfer Learning; ML: Machine Learning; PZ: Peripheral Zone; TZ: Transition Zone; AFS: Anterior Fibromuscolar Stroma.

© 2021 by the authors. Licensee MDPI, Basel, Switzerland. This article is an open access article distributed under the terms and conditions of the Creative Commons Attribution (CC BY) license (http://creativecommons.org/licenses/by/4.0/).
